# Risk factors for *Clostridium difficile* infection in surgical patients hospitalized in a tertiary hospital in Belgrade, Serbia: a case–control study

**DOI:** 10.1186/s13756-017-0188-x

**Published:** 2017-03-27

**Authors:** Vesna Šuljagić, Ivan Miljković, Srđan Starčević, Nenad Stepić, Zoran Kostić, Dragutin Jovanović, Jelena Brusić-Renaud, Biljana Mijović, Sandra Šipetić-Grujičić

**Affiliations:** 1 Department of Nosocomial Infections Control, Military Medical Academy, 11 000 Belgrade, Serbia; 2 Faculty of Medicine of Military Medical Academy University of Defence, 11000 Belgrade, Serbia; 3 Institute of Epidemiology, Military Medical Academy, 11 000 Belgrade, Serbia; 4 Clinic for Orthopedic Surgery and Traumatology, Military Medical Academy, 11 000 Belgrade, Serbia; 5 Clinic for Plastic Surgery and Burns, Military Medical Academy, 11 000 Belgrade, Serbia; 6 Clinic for General Surgery, Military Medical Academy, 11 000 Belgrade, Serbia; 7 Institute of Microbiology Military Medical Academy, 11 000 Belgrade, Serbia; 8 Sector for Pharmacy, Military Medical Academy, 11 000 Belgrade, Serbia; 9 Faculty of Medicine, University of East Sarajevo, 73300 Foča, Republic of Srpska, Bosnia and Herzegovina; 10 Institute of Epidemiology, Faculty of Medicine, University of Belgrade, 11000 Belgrade, Serbia

**Keywords:** *Clostridium difficile*, Incidence, Risk factors, In-hospital mortality, Surgical patients

## Abstract

**Background:**

The objective of this study was to investigate independent risk factors (RFs) connected with healthcare-associated (HA) *Clostridium difficile* infection (CDI) in surgical patients, its frequency per surgical wards and in-hospital-mortality at a single hospital.

**Methods:**

Risk factors for the infection were prospectively assessed among surgical patients with laboratory confirmed HA CDI and compared with a control group without HA CDI.

**Results:**

The overall incidence rate of HA CDI was 2.6 per 10000 patient-days. Significant independent RFs for HA CDI were the use of carbapenems (*P* = 0.007, OR: 10.62, 95% CI: 1.93–58.4), the admission to intensive care unit (*P* = 0.004, OR:3.00, 95% CI:1.41–6.40), and the administration of 3rd generation cephalosporins (*P* = 0.014, OR:2.27, 95% CI:1.18–4.39). Patients with HA CDI had significantly higher in–hospital mortality compared to controls (*P*: 0.007; OR: 8.95; 95% CI: 1.84–43.43).

**Conclusions:**

CDI is an important HA infection in population of surgical patients and this study emphasizes the importance of the wise use of antibiotics, and other infection control strategies in order to prevent HA CDI, and to decrease the incidence and in-hospital mortality rate.

## Background

During the current century the epidemiology of *Clostridium difficile* (*C.difficile*) infections (CDI) has changed rapidly, with increases noted in the incidence of diseases internationally, and in the reports of CDI outbreaks within healthcare institutions in the USA, Canada and Europe. The increase of CDI incidence was partly associated with the epidemic emergence of a new C. difficile ribotype 027, which was also described to cause more severe infections [1]. CDI became one of the most common healthcare-associated (HA) infections in modern medicine. CDI results in a wide spectrum of clinical conditions, including an asymptomatic carrier state; mild, self-limited diarrhea; pseudomembranous colitis; and fulminant colitis. It is associated with the increased morbidity, in-hospital mortality, prolonged hospitalization, and increased costs. The main risk factors for CDI include the administration of antibiotics, patients older than 65 years, multiple co-morbid conditions, previous hospitalization, medical procedures, and long stay in hospitals [2–4]. The reported incidence of HA CDI varies according to the country, the size of institution and ward location, the type of population studied [5–11]. Patients undergoing surgical procedures are at risk of the postoperative HA CDI development. Most studies of risk factors (RFs) for CDI in surgical patients have been conducted in the USA or Western European countries, which are characterized by the well-developed healthcare systems [8–10, 12]. In contrast, the burden of CDI in Serbia, a country in socioeconomic transition and with a resource-limited healthcare system, is less well studied [3, 11]. The burden of CDI in surgical patients in Serbia is unclear.

The aim of this study was to investigate independent RFs associated with HA CDI in surgical patients, its frequency per surgical wards and in-hospital-mortality in a tertiary healthcare centre in Serbia.

## Methods

### Patients and databases

The Military Medical Academy (MMA), Belgrade, Serbia, a teaching hospital of the University of Defense, is a 1200-bed tertiary healthcare centre divided into 27 departments. The department of Infection Control performs continuous surveillance of the MMA patients. The surveillance of HA infections, including CDI, covered patients in surgical clinics of the MMA: Urology, General Surgery, Orthopedics/Traumatology, Neurosurgery, Maxillofacial Surgery, Plastic Surgery/Burns, Vascular Surgery, Thoracic Surgery, Cardio Surgery, Otorhinolaryngology, and Ophthalmology.

Through regular hospital surveillance of surgical patients we prospectively identified all patients who had new HA, laboratory confirmed postoperative CDI during the study period, from 1st January 2011 to 31st December 2012. Reviewing the clinical chart information on patient characteristics, RFs related to healthcare were collected. We gathered data on the following variables: intrinsic factors (existing at admission) sex, age, *Diabetes mellitus*, malignancy, and factors related to healthcare including previous hospitalization in other hospitals, intensive care unit (ICU) admission, duration of treatment in ICU, mechanical ventilation, nasogastric tubes, previous use of corticosteroids, histamine-2-receptor antagonists (H2RAs), proton-pump inhibitors (PPIs) and antibiotics (number, type, and duration of used antibiotics). Also, data about in-hospital mortality were recorded.

In the case–control study, every surgical patient with HA CDI was compared with two control patients without CDI. HA CDI was defined as diarrhea (≥3 daily) which was acquired more than three days after admission to the surgical department and detection of *C. difficile* toxins A and B in a stool sample, which was derived more than three days after admission. Control patients were matched to the cases according to the inpatient ward, gender, age (±5 years), and date of surgical operation.

### Microbiological testing

Microbiological testing was performed at the Institute of Medical Microbiology at the MMA. Enzyme immunoassay kits for *C. difficile* toxins A and B were used (*BIOMERIEUX-VIDAS Clostridium difficile toxins A&B CDAB*). Only patients who had never been diagnosed with CDI at the MMA were included in the control group, and only those who had not been diagnosed with CDI before enrolling in the study, were included in the case group, respectively. The Research Ethics Board of the MMA approved the research protocol. All study participants provided the informed written consent.

### Statistical analysis

Incidence rate (IR) was defined as the number of HA CDIs per 10000 patient-days and per 1000 patients treated. The rate of testing (TR) was defined as the number of *C. difficile* toxin tests performed per 10000 patient-days. The in-hospital mortality rate was defined as the number of deaths per 100 patients. Data analyses were performed with SPSS, version 21.0 (SPSS, Inc, Chicago, IL). Results were expressed as the mean ± SD or as the proportion of the total number of patients. The *χ* ^2^ test or Fischer exact test were used for categorical variables and relative risk, and their corresponding 95% confidence intervals (CI) were calculated. For parametric continuous variables, mean values were compared using the Student *t* test. For nonparametric continuous variables the Mann–Whitney *U* test was used. RF independently associated with CDI were identified by stepwise logistic regression analysis of variables selected by univariate analysis, with a limit for enetering and removing variables from the model at 0.05.

## Results

During 2011–2012 in the MMA 29033 surgical patients were treated during 255431 patient-days. A total of 67 surgical patients with CDI were registered (IR: 2.6 per 10000 patient-days or 2.3 per 1000 patients, TR: 8.5 per 10000 patient-days) (Table 1).

**Table 1:** Incidence rates of *Clostridium difficile* infections in 11 surgical wards of Military Medical Academy during 2011–2012

Ward	Hospital Inpatient Days/Number of patients treated	New cases per 10000 patient-days	New cases per 1000 patients	Testing frequency per 10000 patient-days
Urology	39022/5968	1.5	1.0	6.7
General Surgery	42063/4358	1.7	1.6	6.4
Traumatology/Orthopaedics	44139/3716	6.1	7.2	12.5
Neurosurgery	22546/2639	0	0	4.4
Maxillofacial Surgery	16829/2242	0.6	0.4	2.4
Plastic Surgery and Burns	24019/2414	4.6	4.6	11.2
Vascular Surgery	20175/1631	1.5	1.8	16.9
Thoracic Surgery	12408/1191	0.8	0.8	7.3
Cardiosurgery	13842/827	6.5	10.9	14.5
Otorhinolaryngology	20388/4047	1.0	0.5	2.0
Overall	255431/29033	2.6	2.3	8.5

Demographic and clinical characteristics of patients in the case and control groups according to ULRA are shown in Table 2. MLRA identified three independent RFs associated with CDI in surgical patients: the previous administration of carbapenems (*P* = 0.007, OR:10.62, 95% CI:1.93–58.4), the 3rd generation of cephalosporins (*P* = 0.014, OR:2.27, 95% CI:1.18–4.39) and admission to the ICU (*P* = 0.004, OR:3.00, 95% CI:1.41–6.40). According to the ULRA, PPIs usage was a significant RF for HA CDI (*P* = 0.05), but it was not a significant independent RF according to MLRA (*P* = 0.051, OR:2.7, 95% CI:1.00–7.19).

**Table 2:** Distribution of cases and controls according to their demographic and clinical characteristics: results of univariate analysis

Variable	Case N° (%)	Controls N° (%)	*p* value
	*n =* 67	*n =* 134	
Male	32 (47.8)	64 (47.8)	1.00
Age			
≤ 40	2 (2.9)	5 (3.7)	0.786
41–64	32 (47.8)	58 (43.3)	0.547
≥ 65 years	33 (49.2)	71 (52.9)	0.618
*Diabetes mellitus*	10 (14.9)	15 (11.2)	0.451
Malignacy	14 (20.9)	22 (16.4)	0.436
Previous hospitalization	13 (19.4)	19 (14.1)	0.342
ICU admission	22 (32.8)	19 (14.1)	0.003
≥ 5 days in ICU	67 (100.0)	121 (90.3)	0.051
Nasogastric tube	5 (7.4)	1 (0.7)	0.032
Mechanical ventilation	4 (5.9)	0 (0.0)	0.032
Proton-pump inhibitors	13 (19.4)	8 (5.9)	0.005
H2 receptor antagonist	40 (59.7)	62 (46.2)	0.074
Corticosteroides	4 (5.9)	1 (0.7)	0.059
One antibiotic	32 (47.8)	85 (63.4)	0.035
Days of usage an antibiotic	5.13 ± 2.14	4.40 ± 2.27	0.120
Two antibiotics	20 (29.9)	27 (20.1)	0.128
Days of usage two antibiotics	5.85 ± 3.79	4.63 ± 2.13	0.167
Three antibiotics	9 (13.4)	5 (3.7)	0.017
Days of usage three antibiotics	7.89 ± 5.89	5.60 ± 2.07	0.423
Four antibiotics	4 (6.0)	2 (1.5)	0.103
Days of usage four antibiotics	8.75 ± 4.35	7.00 ± 2.83	0.642
Cephalosporins 1st gen.	15 (22.4)	33 (24.6)	0.726
Days of usage cephalosporins 1st gen.	1.15 ± 2.47	1.04 ± 2.16	0.742
Cephalosporins 2nd gen.	14 (20.9)	25 (18.7)	0.705
Days of usage cephalosporins 2nd gen.	1.18 ± 2.50	0.96 ± 2.25	0.537
Cephalosporins 3rd gen.	42 (62.7)	62 (46.3)	0.029
Days of usage cephalosporins 3rd gen.	4.39 ± 4.59	2.51 ± 3.25	0.001
Aminiglycosides	7 (10.4)	18 (13.4)	0.546
Days of usage Aminoglycosides	0.91 ± 3.58	0.66 ± 2.10	0.528
Quinolones	6 (8.9)	4 (3.0)	0.080
Days of usage Quinolones	0.52 ± 1.76	0.16 ± 0.94	0.056
Sulfonamides	3 (4.5)	5 (3.7)	0.799
Days of usage Sulfonamides	0.25 ± 1.26	0.15 ± 0.86	0.491
Carbapenems	6 (9.0)	2 (1.5)	0.024
Days of usage Carbapenems	0.62 ± 2.10	0.10 ± 0.85	0.014

In hospital-mortality was significantly higher by 9-fold in HA CDI compared to control (12% *vs* 1.5%, *P*: 0.007; OR: 8.95; 95% CI: 1.84–43.43). Of 8 deaths in the case group, 6 or 75% were in the older than 65.

## Discussion

Most of epidemiological data about CDI in surgical patients have been limited to North America and West Europe [8–10, 12]. This study provides data about IR, RF and in-hospital-mortality of HA CDI in surgical patients admitted to the tertiary healthcare centre in the South East Europe.

The overall IR of HA CDI was 2.6 per 10000 patient-days, varied by different wards from 0 in Neurosurgery to more than 6 per 10000 patient-days in Orthopedics/Traumatology and Cardio Surgery. The overall TR was 8.5 per 10000 patient-days varied by different wards from 2.0 in Otorhinolaryngology to 16.9 per 10000 patient-days in Vascular Surgery. Our results of IR were similar to data reported by a coordinating laboratory for Portugal (2.9 for 2011–2012 and 3.0 for 2012–2013), but lower than mean IR of 7.0 of HA CDI (country range 0.7–28.7) in a European, multicentre, prospective, biannual point-prevalence study of CDI in hospitalized patients with diarrhea (EUCLID) (4). In our hospital we have the practice of routine testing of all submitted diarrheal inpatient samples, but our TR in surgical patients in the two-year period was far lower than mean TR in European hospitals (65.8 tests, country range 4.6–223.3) (4).

An association between female gender and CDI has been reported by previous population-based studies from the USA [5]. Similarly to the report of Rodrigues et al. [9], our results showed no significant association between gender and CDI. Advance age is an independent RF for CDI in many studies [5, 9, 10]. RFs specific to older adults are frequent interactions with healthcare systems and age-related changes in physiology, including immune senescence and changes of the gut microbiome [13]. In our study majority of CDI occurred in patients older than 40 years and half in patients older than 65 years. We did not analyze age and sex in the model of MLRA because cases and controls were matched according to those variables.

Among the main findings of this study are the identification of significant independent RFs for CDI in surgical patients, including carbapenems, admission to ICU and the 3rd generation of cephalosporins. The disruption of the normal flora caused by antibiotics allows *C. difficile* to colonize and overgrow within the gastrointestinal tract. Nearly all antibiotics have been implicated in CDI, but certain classes seem to cause higher risk for CDI. Stojanović confirmed in his study that all the groups of antibiotics, except for tetracycline, and trimetoprim- sulfamethoxazole, were statistically significant RFs for CDI [3]. The administration of quinolones emerged as the most important RF for CID in Quebec during an epidemic caused by a hipervirulent strain of *C. difficile* [14]. A recent study from England showed that restricting quinolones prescribing was associated with a decline in incidence of CDI [15]. The majority of patients in our study received at least one class of antibiotic. The third-generation cephalosporins were the most commonly used antibiotics in both groups and an independent RF for CDI. The study of Korean authors showed that the use of cephalosporins increased the risk for CDI [4]. Also, the use of carbapenems, in addition to having the higher frequency in the case group, was an independent RF for CDI in our study. The study of Metzger et al. showed that carbapenems usage was associated with CDI in ULRA, but didn't retain significance as an independent RF in MLRA [16].

The admission to ICU was significantly associated with CDI in our patients as reported before [4, 9]. Critically ill patients in ICU share many of the RFs for developing CDI such as: severe underlying diseases, antibiotic exposure, gastric acid suppression with H2RAs or PPIs, the use of mechanical ventilation, and nasogastric tubes [17].

However, in a meta-analysis, Kwok et al. demonstrated the possible association between the PPI use and the incident and recurrent CDI. This risk was further increased by concomitant use of antibiotics and PPI, whereas H2RAs may be less harmful [18]. In our study, according to ULRA, both groups of patients received H2RAs (59.7% of cases and 46.2% controls) more frequently than PPIs (19.4% and 5.9%), but only PPIs significantly increased the risk of CDI without being a significant independent RF. Also, mice model demonstrated that PPIs administration can increase the severity of CDI induced by an antibiotic cocktail [19]. Medications that suppress gastric acid have been associated with the alteration of gastrointestinal flora and the increased susceptibility to gastrointestinal infections [20].

The mortality rate in patients with CDI in this study was 12.1% and significantly higher than in the control group. Also, it is higher than it was reported in Veterans Health Administration (12.1% *vs* 5.3%) [8]. This difference is explained by the different levels of healthcare and treatment of patients in hospitals in Serbia and the USA.

Of 8 deaths in the case group, 6 or 75% were in the older than 65. CDI was not the primary cause of death but it was mentioned in the clinical chart information. A systematic review of unfavorable outcomes of CDI (68 studies), based on publications from 1978 until 2013, showed that mortality was associated with age, co-morbidities, hypo-albuminemia, leucocytosis, acute renal failure, and infection with ribotype 027 [21].

Last data from a European, multicentre, prospective, biannual point-prevalence study of CDI showed that overall prevalence of ribotype 027 has risen more than three-fold (from 5% to 18%) and high endemicity of ribotype 027 has shifted from the UK and Ireland in 2008, to Germany and Eastern Europe in 2012–13 [6, 7]. The study of Rupnik et al. analyzed PCR ribotype distribution of 249 *C. difficile* isolates received for typing from six hospital settings from South Europe (the MMA was not included) in time period from 2008 to 2015 and showed that PCR ribotype 027 and related ribotype 176 were detected in outbreaks in Croatia, Bosnia and Herzegovina and Serbia [22]. Some PCR ribotypes are more often associated with severe diseases and/or are easily transmitted and linked to outbreaks. In our study we did not know if all those cases were from the outbreak because of the inability of laboratory pathogen isolation and the impossibility of determining its ribotype.

Important limitation of this study is that CDI testing was based on toxin A/B enzyme immunoassay (EIA) as the only diagnostic procedure in laboratory (no EIA detecting glutamate dehydrogenase, no nucleic acid amplification tests, no isolation of *C. difficile* and detection of toxigenic isolates). This procedure has shown poor sensitivity of less than 50% in studies of Shin [23] and Swindells [24]. The meta-analysis of Crobach et al. showed that no single test can be used as a stand-alone test for diagnosed CDI as a result of inadequate positive predictive values at low CDI prevalence [25]. *C. difficile* toxins can degrade at room temperature and the quality of CDI diagnostic also depends on transport time of the samples in the respective settings of the MMA. Both, the low rate of test performance in general and limitations of diagnostic test system, which we used, make it very likely that the actual incidence of CDI in our study was markedly higher.

Another limitation is the possibility of confounding variables that were not examined in our study. Although confounding variables were chosen after an exhaustive search of the literature, the potential for oversight and exclusion does exist. We tried to find all of the data in order to gain an understanding of the RF; we did not include some parameters, namely the diagnosis upon admission, all comorbidities, durations of the hospital stays, PPIs exposure in previous 30 days, the duration of PPIs and H2RA use, and outpatient antibiotic use, and analyzing these factors could have enhanced the relevance of our results. Furthermore, we did not evaluate the CDI cases and the controls in relation to the sex, age and wards in which they had undergone treatment, because CDI cases and controls were matched according to them.

Another limitation is that we performed case–control study and that we have small sample size.

The strengths of our study include its setting in a teaching hospital, and its 2-year duration. ULRA and MLRA strengthened the evidence.

## Conclusions

The results of the present study are valuable in documenting the relations between RFs and CDI in patients undergoing surgery and contribute to improve quality of health care. CDI is an important HA infection in population of surgical patients and this study emphasizes the importance of the wise use of antibiotics, and other infection control strategies to prevent HA CDI, decrease incidence and in- hospital mortality rate.

## Abbreviations

*C.difficile*: *Clostridium difficile*
CDI: *Clostridium difficile* infection
CI: Confidence intervals
H2RAs: Histamine-2-receptor antagonists
HA: Healthcare-associated
ICU: Intensive care unit
IR: Incidence rate
MLRA: Multivariate logistic regression analysis
MMA: Military Medical Academy
PPIS: Proton-pump inhibitors
RF: Risk factors
TR: Testing rate

## References

[1] Freeman J, Bauer MP, Baines SD, Corver J, Fawley WN, Goorhuis B (2010). The changing epidemiology of Clostridium difficile infections. Clin Microbiol Rev.

[2] Magee G, Strauss ME, Thomas SM, Brown H, Baumer D, Broderick KC (2015). Impact of Clostridium difficile-associated diarrhea on acute care length of stay, hospital costs, and readmission: A multicenter retrospective study of inpatients, 2009–2011. Am J Infect Control.

[3] Stojanović P (2016). Analysis of risk factors and clinical manifestations associated with Clostridium difficile disease in Serbian hospitalized patients. Braz J Microbiol.

[4] Cho SM, Lee JJ, Yoon HJ (2012). Clinical risk factors for Clostridium difficile-associated diseases. Braz J Infect Dis.

[5] Lessa FC, Mu Y, Bamberg WM, Beldavs ZG, Dumyati GK, Dunn JR (2015). Burden of Clostridium difficile infection in the United States. N Engl J Med.

[6] Davies KA, Longshaw CM, Davis GL, Bouza E, Barbut F, Barna Z (2014). Underdiagnosis of Clostridium difficile across Europe: the European, multicentre, prospective, biannual, point-prevalence study of Clostridium difficile infection in hospitalised patients with diarrhoea (EUCLID). Lancet Infect Dis.

[7] Bauer MP, Notermans DW, van Benthem BH, Brazier JS, Wilcox MH, Rupnik M, ECDIS Study Group (2011). Clostridium difficile infectioninEurope: a hospital-based survey. Lancet.

[8] Li X, Wilson M, Nylander W, Smith T, Lynn M, Gunnar W (2015). Analysis of Morbidity and Mortality Outcomes in Postoperative Clostridium difficile Infection in the Veterans Health Administration. JAMA Surg.

[9] Rodrigues MA, Brady RR, Rodrigues J, Graham C, Gibb AP (2010). Clostridium difficile infection in general surgery patients; identification of high-risk populations. Int J Surg.

[10] Abdelsattar ZM, Krapohl G, Alrahmani L, Banerjee M, Krell RW, Wong SL (2015). Postoperative Burden of Hospital-Acquired Clostridium difficile Infection. Infect Control Hosp Epidemiol.

[11] Šuljagić V, Đorđević D, Lazić S, Mijović B (2013). Epidemiological characteristics of nosocomial diarrhea caused by *Clostridium difficile* in a tertiary level hospital in Serbia. Srp Arh Celok Lek.

[12] Skovrlj B, Guzman JZ, Silvestre J, Al Maaieh M, Quereshi SA (2014). *Clostridium difficile* colitis in patients undergoing lumbar spine surgery. Spine.

[13] Jump RL (2013). Clostridium difficile infection in older adults. Aging health.

[14] Pépin J, Saheb N, Coulombe MA, Alary ME, Corriveau MP, Authier S (2005). Emergence of fluoroquinolones as the predominant risk factor for Clostridium difficile–associated diarrhea: a cohort study during an epidemic in Quebec. Clin Infect Dis.

[15] Dingle KE, Didelot X, Quan TP, Eyre DW, Stoesser N, Golubchik T, et al. Effects of control interventions on Clostridium difficile infection in England: an observational study. Lancet Infectious Diseases. 2017 10.1016/S1473-3099(16)30514-X PMC5368411 28130063

[16] Metzger R, Swenson BR, Bonatti H, Hedrick TL, Hranjec T, Popovsky KA (2010). Identification of risk factors for the development of Clostridium difficile-associated diarrhea following treatment of polymicrobial surgical infections. Ann Surg.

[17] Riddle DJ, Dubberke ER (2009). Clostridium difficile infection in the intensive care unit. Infect Dis Clin North Am.

[18] Kwok CS, Arthur AK, Anibueze CI, Singh S, Cavallazzi R, Loke YK (2012). Risk of Clostridium difficile infection with acid suppressing drugs and antibiotics: meta-analysis. Am J Gastroenterol.

[19] Hung YP, Ko WC, Chou PH, Chen YH, Lin HJ, Liu YH (2015). Proton pump inhibitor exposure aggravates *Clostridium difficile* associated colitis: evidences from a mouse model. J Infect Dis.

[20] Williams C (2001). Occurrence and significance of gastric colonization during acid-inhibitory therapy. Best Pract Res Clin Gastroenterol.

[21] Chakra CN, Pepin J, Sirard S, Valiquette L (2014). Risk factors for recurrence, complications and mortality in Clostridium difficile infection: a systematic review. PLoS One.

[22] Rupnik M, Andrasevic AT, Dokic ET, Matas I, Jovanovic M, Pasic S (2016). Distribution of Clostridium difficile PCR ribotypes and high proportion of 027 and 176 in some hospitals in four South Eastern European countries. Anaerobe.

[23] Shin S, Kim M, Kim M, Lim H, Kim H, Lee K (2012). Evaluation of the Xpert Clostridium difficile assay for the diagnosis of Clostridium difficile infection. Ann Lab Med.

[24] Swindells J, Brenwald N, Reading N, Oppenheim B (2010). Evaluation of diagnostic tests for Clostridium difficile infection. J Clin Microbiol.

[25] Crobach MJ, Planche T, Eckert C, Barbut F, Terveer EM, Dekkers OM, Wilcox MH, Kuijper EJ (2016). European Society of Clinical Microbiology and Infectious Diseases: update of the diagnostic guidance document for Clostridium difficile infection. Clin Microbiol Infect.

